# Supplementary material for: In vivo CRISPR screens reveal SCAF1 and USP15 as drivers of pancreatic cancer
Source: Nat Commun. 2024 Jun 20;15:5266. doi: 10.1038/s41467-024-49450-3 (PMC11189927; doi:10.1038/s41467-024-49450-3)
Supplement: Supplementary file 1 — Supplementary Information [file 41467_2024_49450_MOESM1_ESM.pdf]

Supplementary Information.

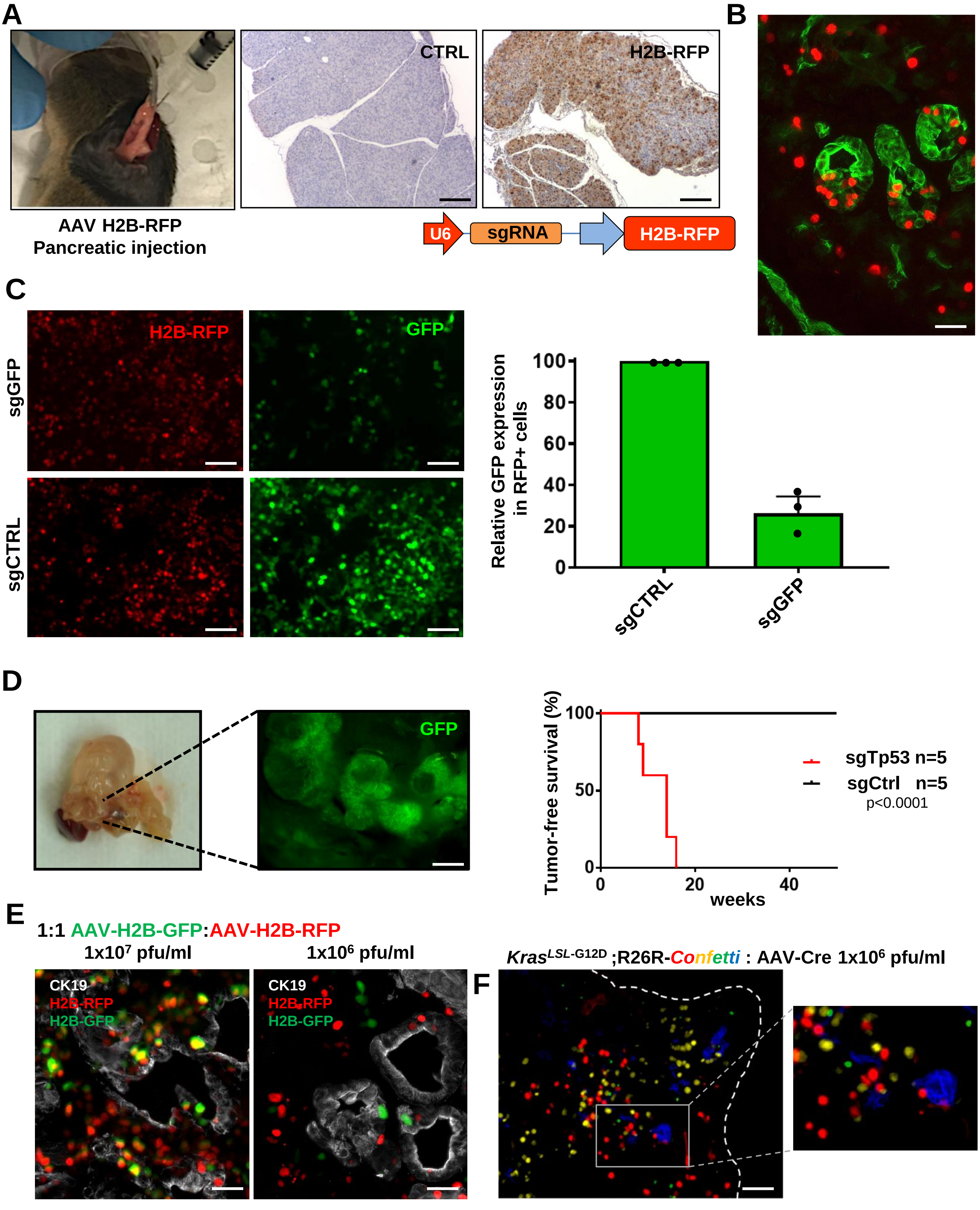

Supplementary Figure 1. ***In vivo*** **CIRPSR knock out efficiency in murine pancreas.**

Supplementary Figure 1. *In vivo* CIRPSR knock out efficiency in murine pancreas. A, Image of AAV injection into the pancreas. Representative immunohistochemistry of pancreas injected with control or AAV H2B-RFP. **B,** Representative immunofluorescence of Pdx1-Cre; LSL-KRas^{G12D} epithelial cells transduced with AAV H2B-RFP. Scale bar 50 µm. **C,** Representative images showing GFP and H2B-RFP expression in Pdx1-Cre;R26-LSL-Cas9-GFP cells transduced with sgGFP-H2B-RFP AAV or control non-targeting sgCTRL-H2B-RFP AAV. Bar graph showing the percent of GFP+/H2B-RFP+ double cells analysed using flow cytometry. Two sided T-test **D** Representative image of a Pdx1-Cre;LSL-KRas^{G12D};R26-LSL-Cas9-GFP pancreas injected with AAV-sgTp53. Tumor-free survival of PDX1-Cre;LSL-KRas^{G12D};LSL-Cas9-GFP mice transduced with a sgTp53 or sgCtrl. Log-Rank test (Mantel-Cox)**E,** Representative images of a pancreas transduced with an AAV-GFP/AAV-RFP 1:1 mixture showing cells transduced with GFP or RFP or double-transduced GFP+/RFP+ cells. The percentage of double positive cells increases at higher viral titre. **F,** Representative image of a reporter LSL-KRas^{G12};R26-LSL-Confetti pancreas infected with AAV-sgRNA-Cre.

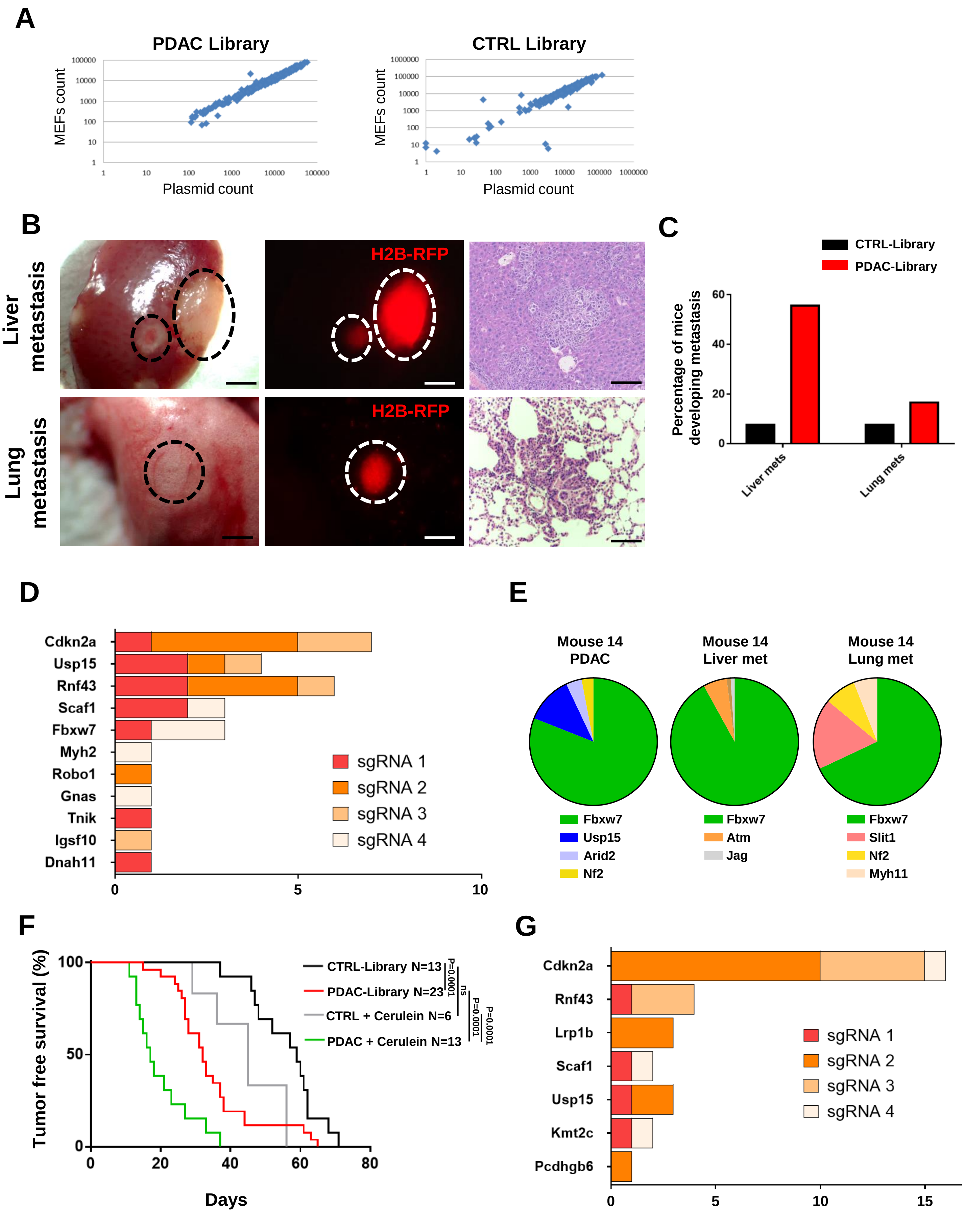

Supplementary Figure 2. *In vivo* CRISPR screen

Supplementary Figure 2. *In vivo* pancreatic cancer CRISPR screen A. Graph showing sgRNA correlation and representation for PDAC and CTRL libraries in plasmid DNA versus transduced MEFs DNA. Each dot represents a guide. **B.** Representative whole mount and immunofluorescence of PDAC-Library liver and lung metastasis showing H2B-RFP expression. Scale bar 3mm. Representative H&E images showing PDAC-Library liver and lung metastasis. Scale bar 100µm. **C.** Percentage of Pdx1-Cre;LSL-*KRas*^{G12D};LSL-*Cas9-GFP* mice transduced with indicated sgRNA library with metastatic disease. **D.** Bar graph showing putative tumor suppressor genes with sgRNAs enriched in metastatic lesions in the PDAC mouse model. **E.** Representative pie charts showing tumor suppressor genes with enriched sgRNAs in tumor DNA obtained from matched pancreatic tumor, liver and lung metastasis. **F.** Tumor-free survival of Pdx1-Cre;LSL-*KRas*^{G12D};LSL-*Cas9-GFP* mice transduced with the PDAC or CTRL library and treated with cerulein. Log-Rank test (Mantel-Cox)**G.** Bar graph showing putative tumor suppressor genes with enriched sgRNAs in tumor DNA obtained from cerulein PDAC mouse model.

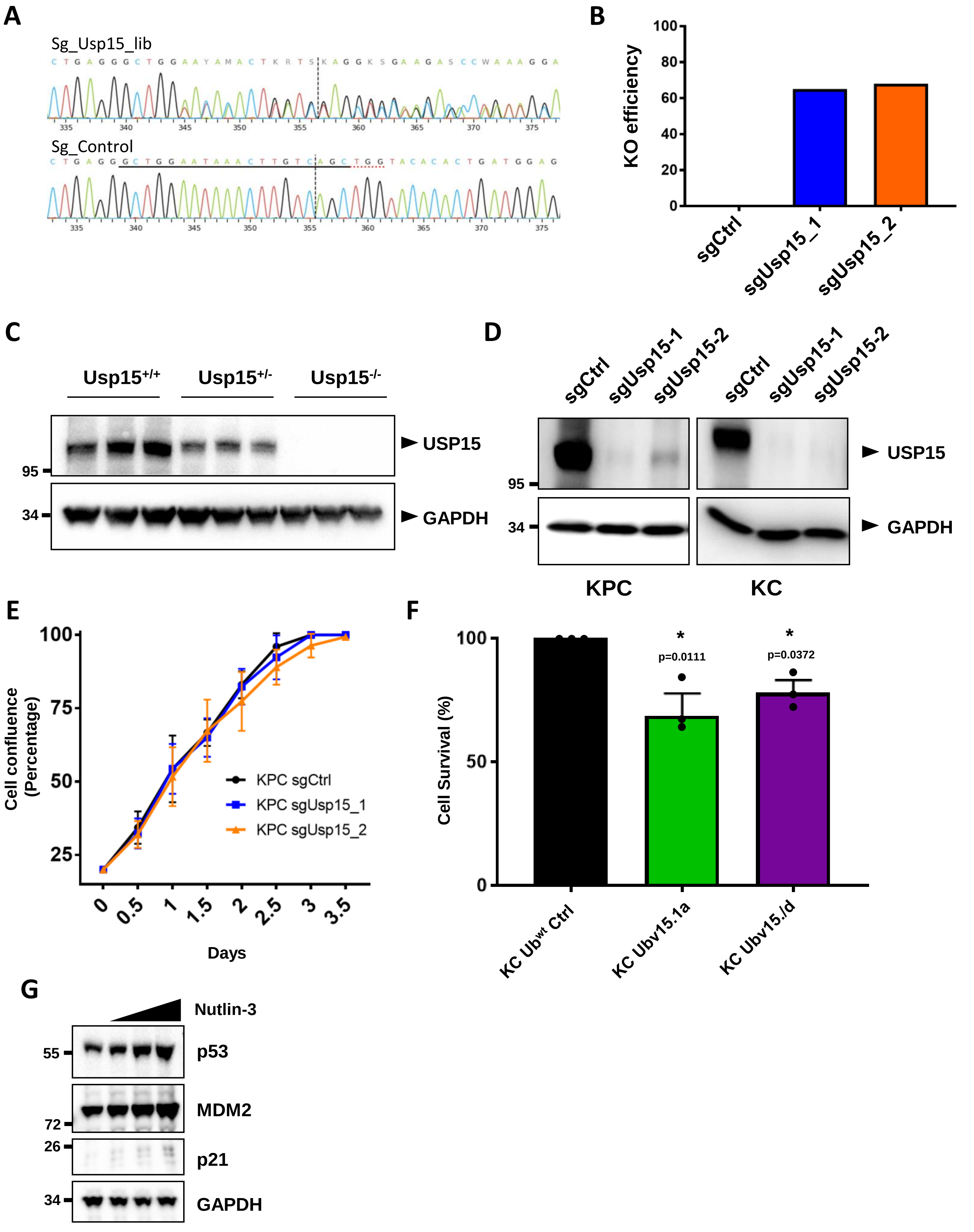

Supplementary Figure 3. ***USP15*** **is a** ***bona-fide*** **PDAC suppressor**

Supplementary Figure 3. *USP15* is a *bona-fide* PDAC suppressor

A. Representative sanger sequencing chromatogram of the DNA sequence from a sgUsp15-targeted sample compared to a control sample. **B.** Gene editing efficiency of sgUsp15. Efficiency was determined using sanger-sequencing data of PCR-amplified sgRNA target sites followed by Tracking of Indels by Decomposition (TIDE https://tide.nki.nl) algorithm on PDAC cells. **C.** Western blot analysis showing expression of Usp15 in Pdx1-Cre;LSL-*Kras*^{G12D} mice with the indicated *Usp15* genotype. **D.** Western blot analysis showing loss of Usp15 protein expression after CRISPR-mediated knockout of Usp15 (two independent sgRNA in KPC cells). **E.** Cell growth curves of KPC cells transduced with sgCtrl or sgUsp15. Data are expressed as cell confluence percentage (%; mean ± SD, n = 3 independent experiments). Two-way ANOVA, Dunnett's multiple comparison **F,** Cell survival of KC cells expressing different ubiquitin variants treated with 4µM of Olaparib (%; mean ± SD, n = 3 independent experiments). Cell percentage normalized to control. Two-sided T-test **G,** Western blot analysis of p53, MDM2 and p21 in KC cells upon treatment with increasing concentrations of nutlin-3 (5, 10, 20 µM; for 24 h).

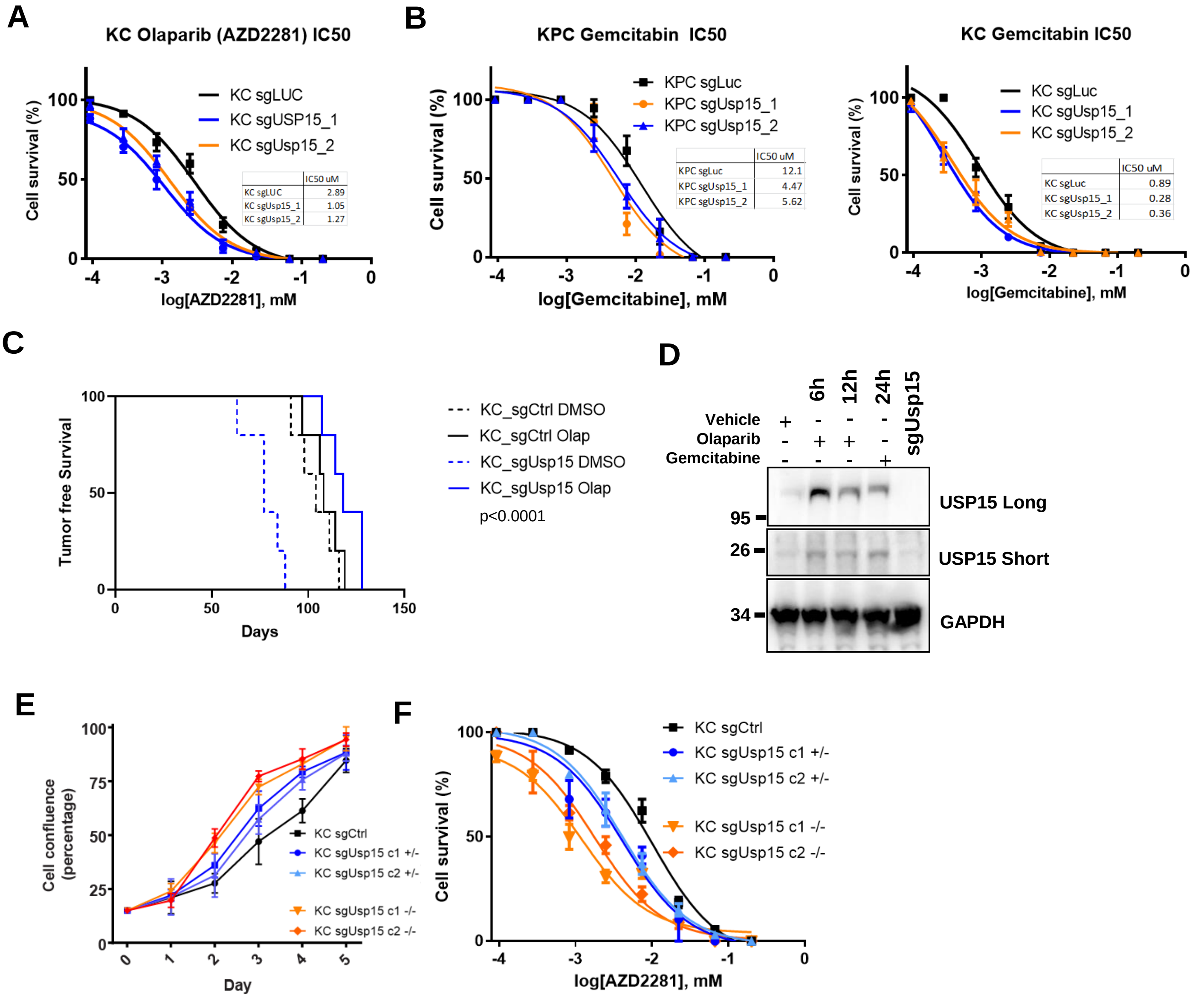

Supplementary Figure 4. ***USP15*** **regulates response to PARPi and Gemcitabine**

Supplementary Figure 4. *USP15* regulates response to PARPi and Gemcitabine
A. Dose-response curves for KC sgCtrl or sgUsp15 cells treated with the indicated concentration of olaparib in cell proliferation assay (%; mean ± SD, n = 3 independent experiments). Two-way ANOVA, Dunnett's multiple comparison **B.** Dose-response curves for KPC and KC sgCtrl or sgUsp15 cells treated with the indicated concentration of Gemcitabine in cell proliferation assay (%; mean ± SD, n = 3 independent experiments). Two-way ANOVA, Dunnett's multiple comparison **C.** Tumor-free survival of NSG mice orthotopically injected with sgCtrl or sgUsp15 KC cells treated daily with vehicle (DMSO) or olaparib (50 mg/kg; i.p.; 5 days on/2 days off). Log-Rank test (Mantel-Cox) **D.** Western blot analysis of USP15 isoform expression in KC cells treated with the indicated drug. **E.** Cell proliferation curves of clonal KC cells with the indicated genotype shown as percentage of cell confluence (%; mean ± SD, n = 3 independent experiments). Two-way ANOVA, Dunnett's multiple comparison **F,** Dose-response curves for clonal KC cells treated with the indicated concentration of olaparib in cell proliferation assay (%; mean ± SD, n = 3 independent experiments). Two-way ANOVA, Dunnett's multiple comparison.

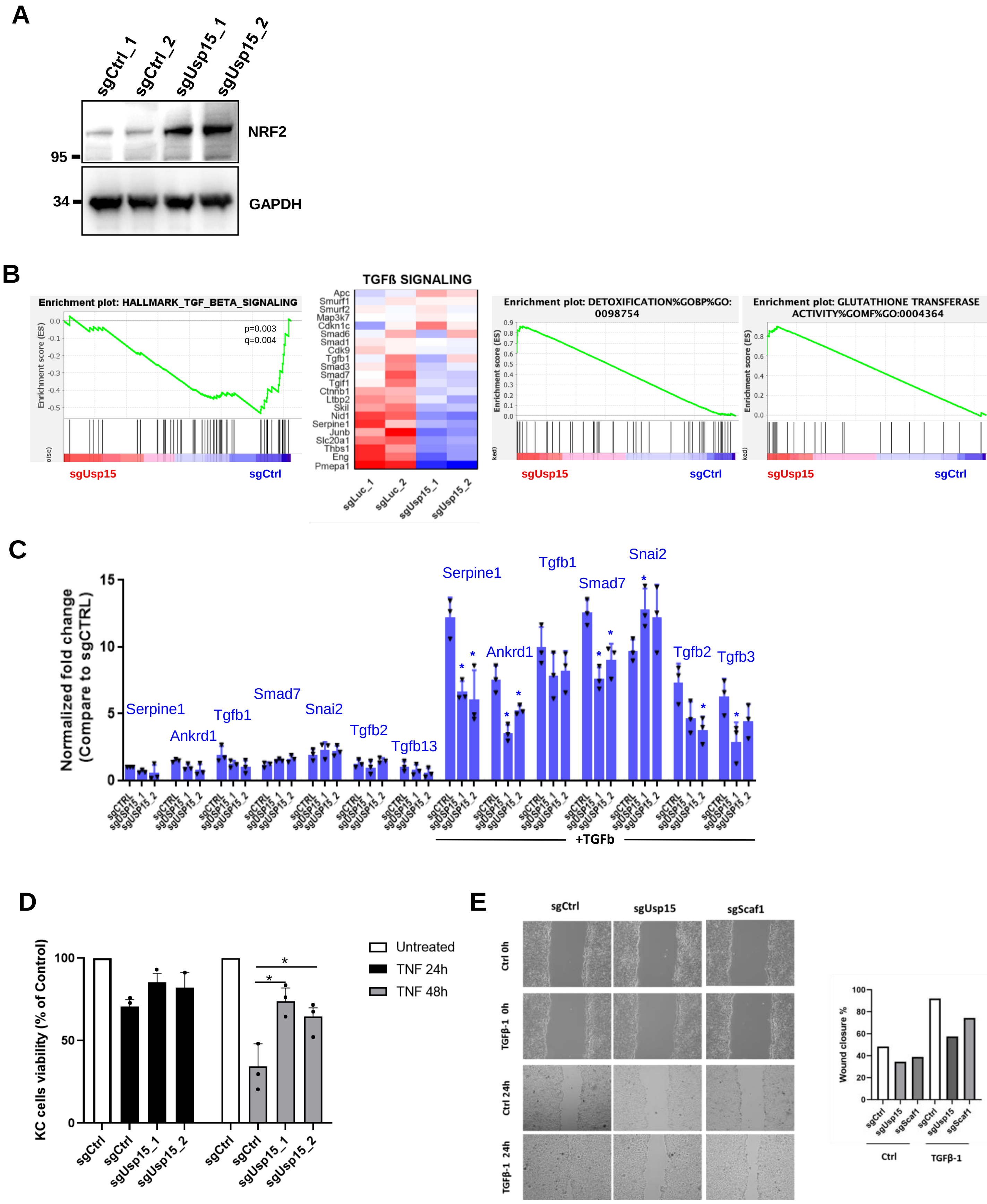

Supplementary Figure 5. *USP15* regulates NRF2, TGFb and TNFa signaling

Supplementary Figure 5. ***USP15 regulates NRF2, TGFb and TNFa signalling***

A, Western blot analysis of NFR2 expression in KC cells transduced with AAV-sgCtrl or AAV-sgUsp15. **B,** GSEA plots and Heatmap of log2 counts per million for TGFβ related genes in KC cells. GSEA enrichment plots of the indicated differentially expressed pathways associated with loss of Usp15. **C,** Expression levels of genes related to TGFβ signalling assessed by RT-qPCR. Results were normalized with Gapdh and are expressed in fold change to CTR (mean ± SEM, n = 3 independent experiments). Cells were incubated with 10 ng/mL TGFβ -for 30 min. Two-sided T-test (Serpine1 p=0.021/p=0.034;Ankrd1 p=0.028/p=0.037; Smad7 p=0.037/p=0.042; Snail2 p=0.046; Tgfb2 p=0.042/p=0.033; Tgfb3 p=0.047).**D,** TNF-α induced cell death is mediated by USP15. KC sgCtrl and sgUsp15 cells were treated with 100 ng/ml TNF-α for the indicated time and cell viability was determined by PrestoBlue™ Cell Viability Reagent. (mean ± SEM, n = 3 independent experiments). Two-sided T-test (p=0.0336) **E**, TGF-b1-induced migration depends on USP15. Migration of KC sgCtrl, sgUsp15 and sgScaf1 cells treated with TGF-b1 (10 ng/ml; 24h) was assessed using a wound healing scratch assay. Phase contrast microscopy images of the cells were taken at 0h and 24h. Wound healing closure was quantified as a percentage of the remaining wound area relative to each initial wound area after 24h using imageJ.

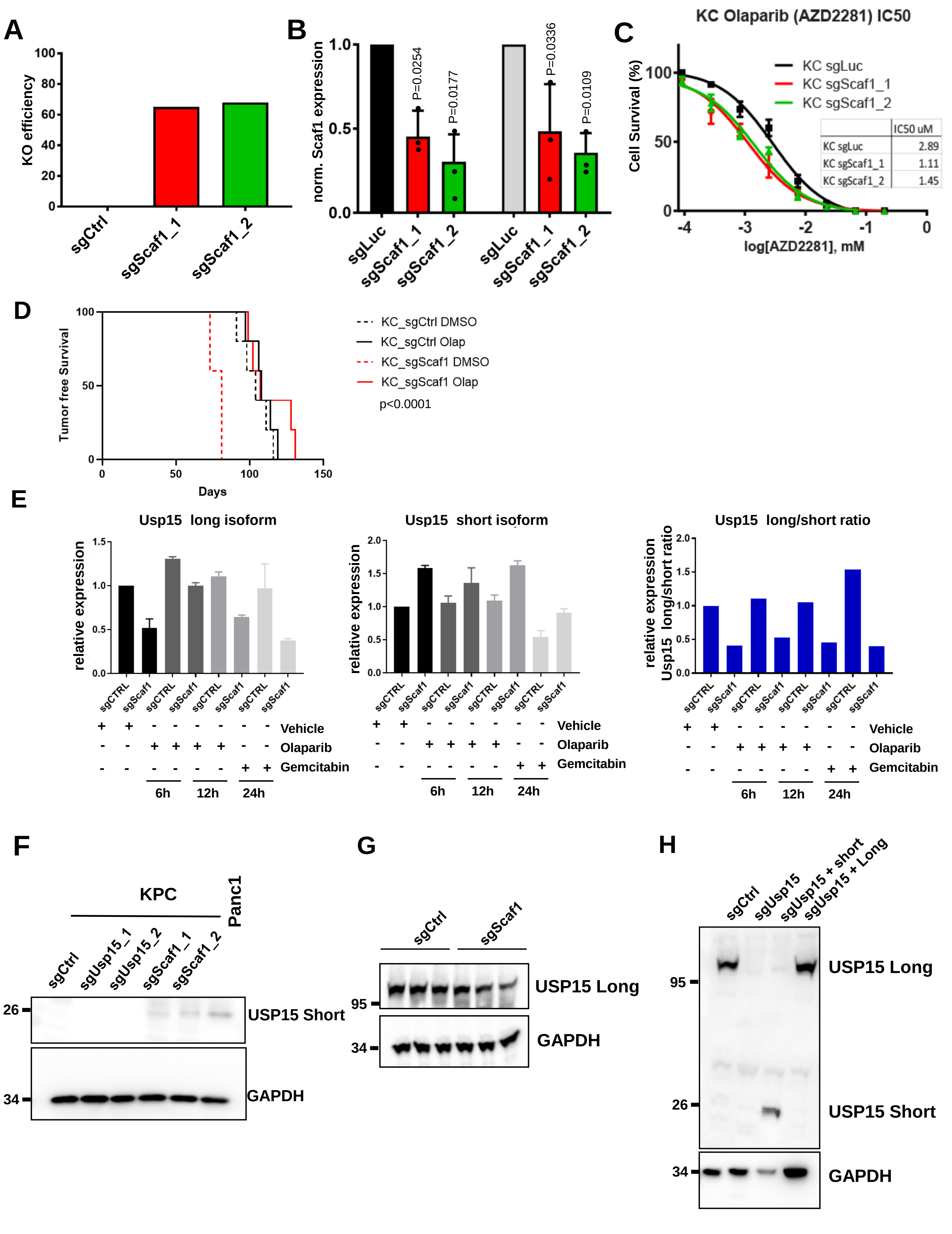

Supplementary Figure 6. *SCAF1* is a bona-fide PDAC suppressor

Supplementary Figure 6. *SCAF1* is a *bona-fide* PDAC suppressor

A. Gene editing efficiency of sgRNAs targeting Scaf1. **B.** RT-PCR analysis of Scaf1 expression in KPC and KC cells transduced with the indicated sgRNAs. **C.** Dose-response curves for KC sgCtrl or sgScaf1 cells treated with the indicated concentration of Olaparib in cell proliferation assay (%; mean ± SD, n = 3 independent experiments). two-way ANOVA, Dunnett's multiple comparison **D.** Tumor-free survival of NSG mice after orthotopic injection of sgCtrl or sgScaf1 KC cells treated daily with vehicle (DMSO) or olaparib. (50 mg/kg; i.p.; 5 days on/2 days off) Log-Rank test (Mantel-Cox) **E.** Quantification of Usp15 long and short isoform expression levels in cells transduced with the indicated sgRNAs and treated with the listed drugs (n=3, mean ± SD) $P \leq 0.05$ **F.** Western blot analysis of short USP15 expression in KPC cells transduced with AAV-sgCtrl, AAV-sgUsp15 or AAV-sgScaf1. Human Panc1 cells are shown as a control. **G.** Western blot analysis showing expression of Usp15 in whole tumor extract of sgCtrl and sgScaf1 mice. **H.** Western blot analysis of USP15 isoform expression in KC cells transduced with the indicated sgRNAs and USP15 expression constructs.

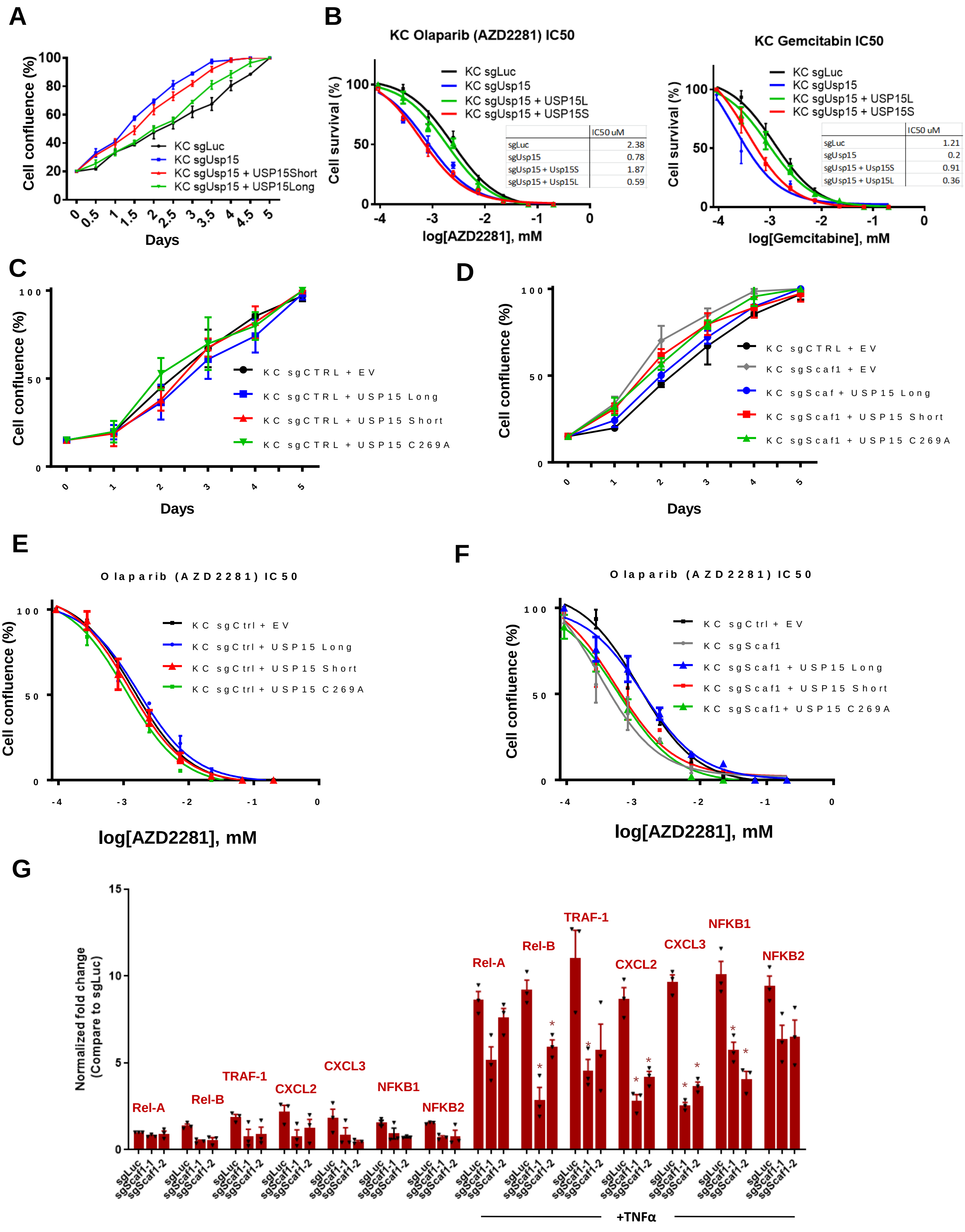

Supplementary Figure 7. ***SCAF1*** **is a** ***bona-fide*** **PDAC suppressor**

Supplementary Figure 7. *SCAF1* is a *bona-fide* PDAC suppressor

A, Cell growth curves of KC cells transduced with the indicated sgRNAs and USP15 expression constructs. Data are expressed as cell confluence percentage (%; mean ± SD, n = 3 independent experiments). Two-way ANOVA, Dunnett's multiple comparison **B,** Dose-response curves of KC cells transduced with the indicated sgRNAs and USP15 expression constructs and treated with Gemcitabine or olaparib in cell proliferation assay (%; mean ± SD, n = 3 independent experiments). Two-way ANOVA, Dunnett's multiple comparison **C.** Cell growth curves of KC transduced with the indicated sgRNA and USP15 expression constructs. Data are expressed as cell confluence percentage (%; mean ± SD, n = 3 independent experiments). Two-way ANOVA, Dunnett's multiple comparison **D.** Cell growth curves of KC transduced with the indicated sgRNAs and USP15 expression constructs. Data are expressed as cell confluence percentage (%; mean ± SD, n = 3 independent experiments). Two-way ANOVA, Dunnett's multiple comparison **E.** Dose-response curves of KC cells transduced with the indicated sgRNAs and USP15 expression constructs and treated with olaparib in cell proliferation assay (%; mean ± SD, n = 3 independent experiments). Two-way ANOVA, Dunnett's multiple comparison **F.** Dose-response curves for KC cells transduced with the indicated sgRNAs and USP15 expression constructs and treated with olaparib in cell proliferation assay (%; mean ± SD, n = 3 independent experiments). Two-way ANOVA, Dunnett's multiple comparison **G,** Expression levels of genes related to TNFa signaling assessed by RT-qPCR. Results were normalized with Gapdh and are expressed in fold change to CTR (mean ± SEM, n = 3 independent experiments). Cells were incubated with 10 ng/mL TGFβ -for 30 min. Two-tailed T-test (Rel-B p=0.027/p=0.034 TRAF-1 p=0.031; CXCL2 p=0.022/p=0.029; CXCL3 p=0.018/p=0.021 ; NFKB1 p=0.031/p=0.026).

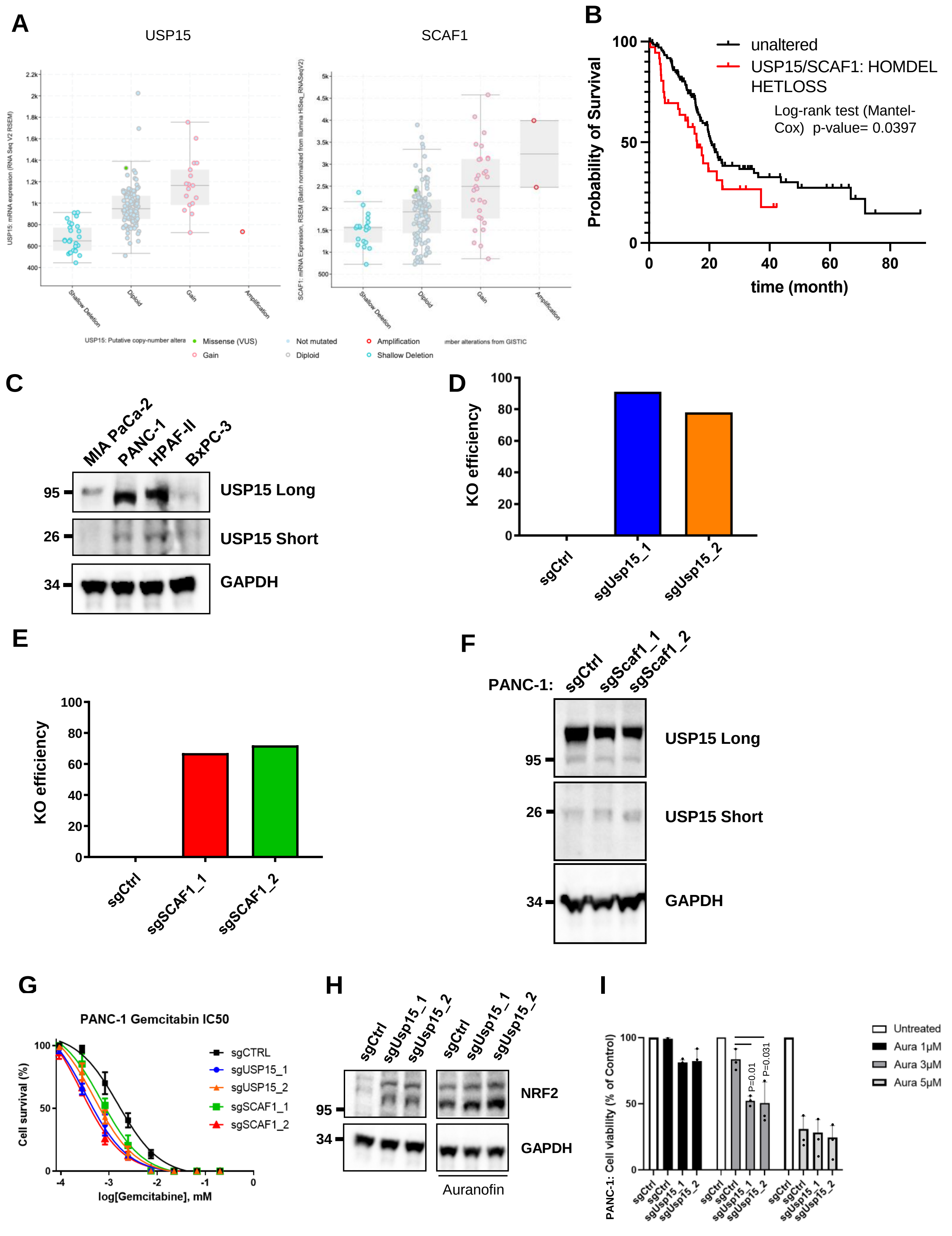

Supplementary Figure 8. *USP15* and *SCAF1* in human cancers

Supplementary Figure 8. *USP15* and *SCAF1* in human cancers
A, USP15 and SCAF1 mRNA expression in human PDAC samples (n= 293, TCGA) according to putative copy number alteration. **B,** Kaplan-Meier survival analyses of PDAC patients with deep or shallow *USP15* or *SCAF1* deletion considered as a group (*37% of patients*, n= 293, TCGA). Log-Rank test (Mantel-Cox) **C,** Western blot analysis showing USP15 long and short isoforms in MIA PaCa-2, PANC-1, HPAF-II and BxPC-3 human PDAC cell lines. **D,** Gene editing efficiency of sgUSP15 in PANC-1 cells. **E,** Gene editing efficiency of sgSCAF1 in PANC-1 cells. **F,** Western blot analysis showing USP15 long and short isoforms in PANC-1 sgScaf1 cells. **G,** Dose-response curves for sgCtrl, sgUsp15 or sgSCAF1 PANC-1 cells treated with Gemcitabin (%; mean ± SD, n = 3 independent experiments). two-way ANOVA, Dunnett's multiple comparison **H,** Western blot analysis showing NFR2 expression in KC sgCtrl and sgUsp15 upon Auranofin treatment (3µM; 24h) I. KC sgCtrl and sgUsp15 cells were treated with indicated concentration of Auranofin for 24h. Cell survival was determined by PrestoBlue™ Cell Viability Reagent (mean ± SD, n = 3 independent experiments) Two-tailed T-test.

A

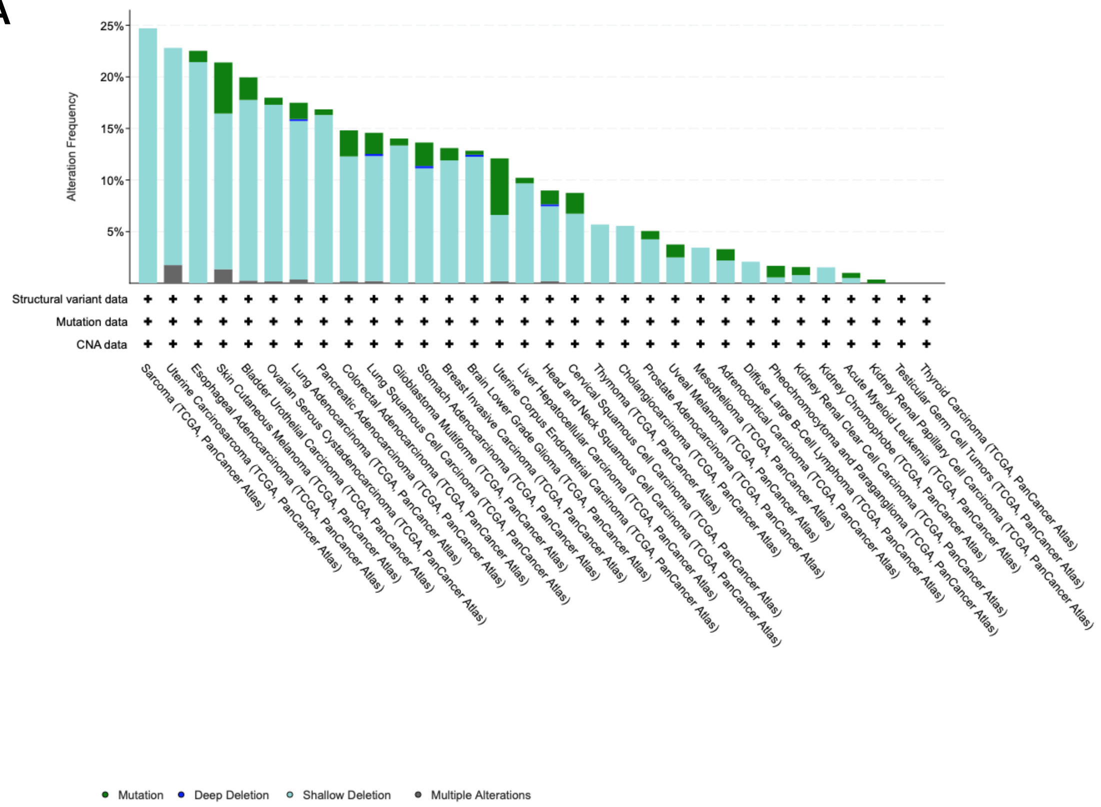

Supplementary Figure 9. ***USP15*** **and** ***SCAF1*** **in human cancers**

Supplementary Figure 9. *USP15* and *SCAF1* in human cancers
A. Bar graph showing *USP15* alteration frequency in other cancers (TCAG).

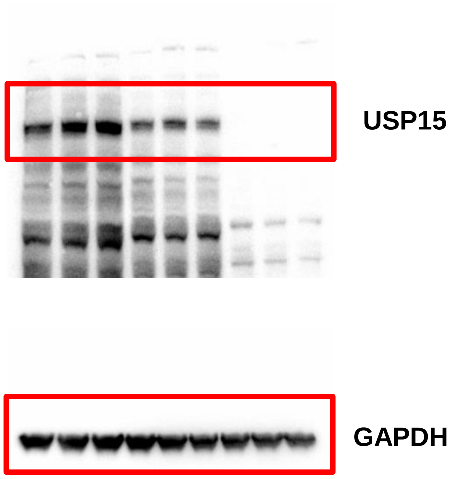

Supp Fig 2.C

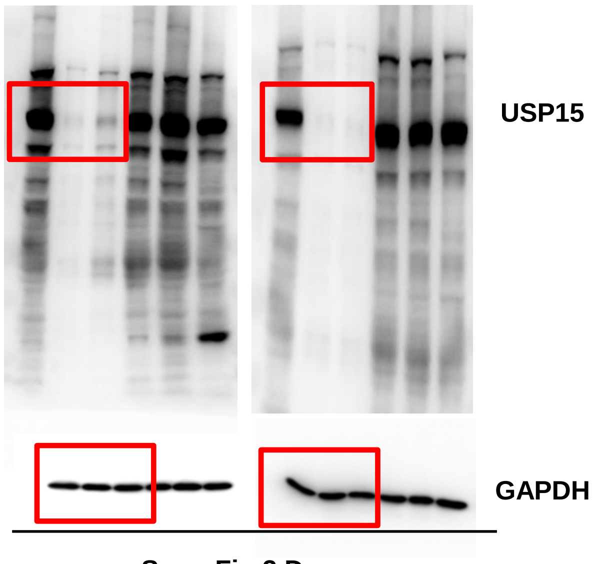

Supp Fig 2.D

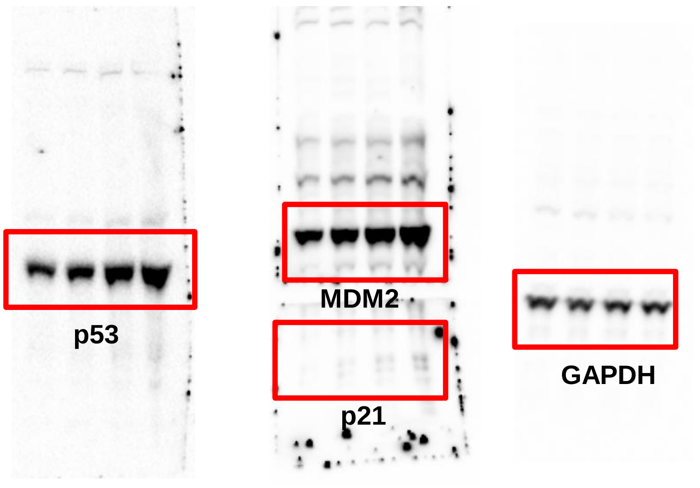

Supp Fig 2.F

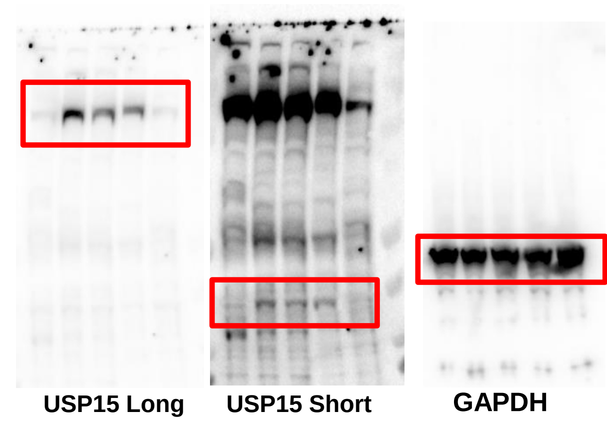

Supp Fig 4.D

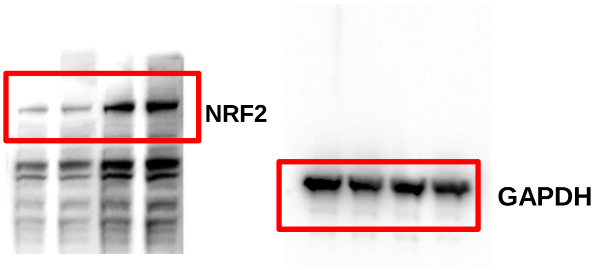

Supp Fig 5.A

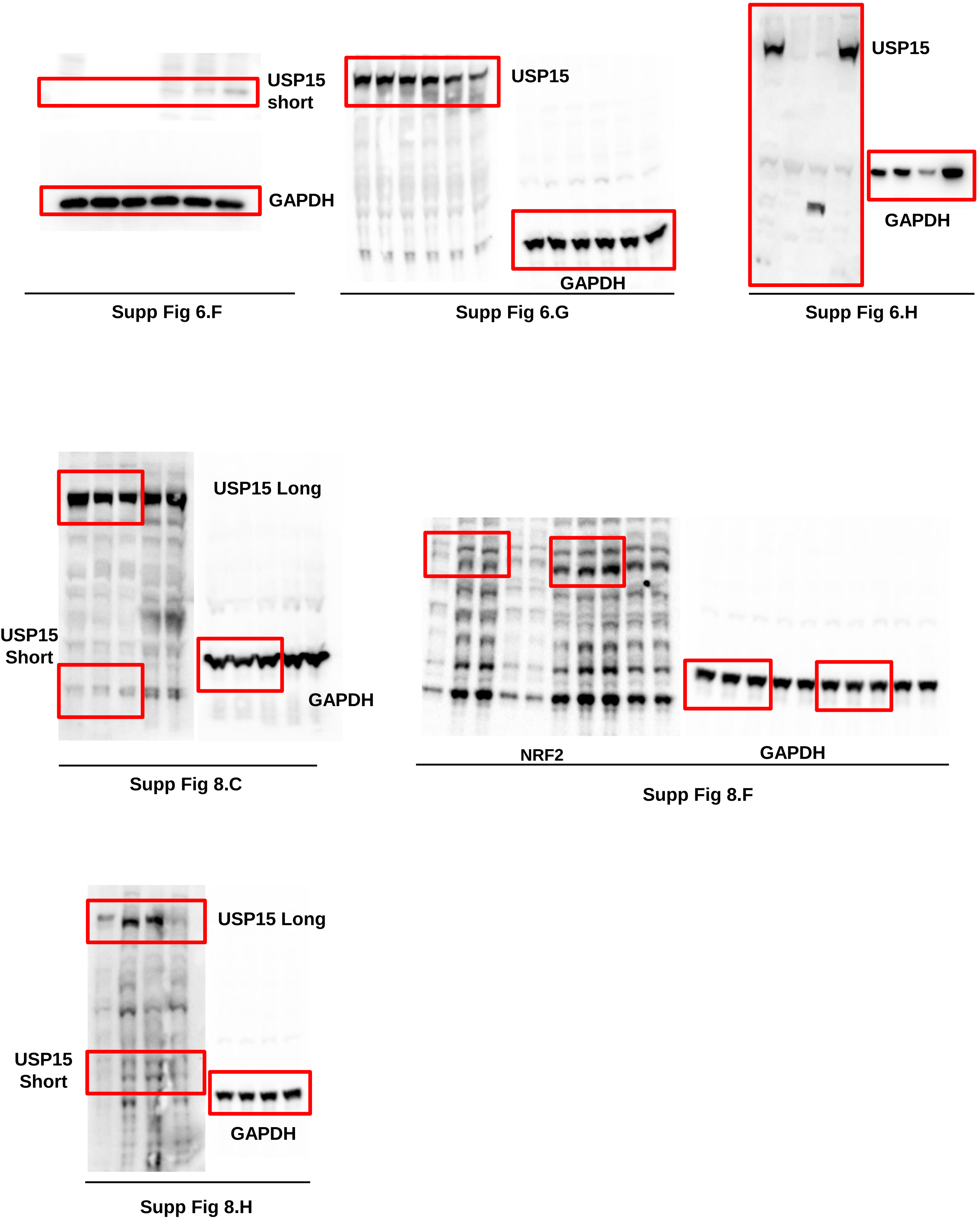

Supplementary Figure 10. *Uncropped scans of western blots*
