# Supplementary material for: In vivo CRISPR screens reveal SCAF1 and USP15 as drivers of pancreatic cancer
Source: Nat Commun. 2024 Jun 20;15:5266. doi: 10.1038/s41467-024-49450-3 (PMC11189927; doi:10.1038/s41467-024-49450-3)
Supplement: Supplementary file 2 — Reporting Summary [file 41467_2024_49450_MOESM2_ESM.pdf]

nature portfolio

Corresponding author(s): Daniel Schramek

Last updated by author(s): Dec 12, 2023

Reporting Summary

Nature Portfolio wishes to improve the reproducibility of the work that we publish. This form provides structure for consistency and transparency in reporting. For further information on Nature Portfolio policies, see our Editorial Policies and the Editorial Policy Checklist.

Statistics

For all statistical analyses, confirm that the following items are present in the figure legend, table legend, main text, or Methods section.

n/a	Confirmed	
☐	☒	The exact sample size (*n*) for each experimental group/condition, given as a discrete number and unit of measurement
☐	☒	A statement on whether measurements were taken from distinct samples or whether the same sample was measured repeatedly
☐	☒	The statistical test(s) used AND whether they are one- or two-sided<br>*Only common tests should be described solely by name; describe more complex techniques in the Methods section.*
☒	☐	A description of all covariates tested
☒	☐	A description of any assumptions or corrections, such as tests of normality and adjustment for multiple comparisons
☒	☐	A full description of the statistical parameters including central tendency (e.g. means) or other basic estimates (e.g. regression coefficient) AND variation (e.g. standard deviation) or associated estimates of uncertainty (e.g. confidence intervals)
☒	☐	For null hypothesis testing, the test statistic (e.g. *F*, *t*, *r*) with confidence intervals, effect sizes, degrees of freedom and *P* value noted<br>*Give P values as exact values whenever suitable.*
☒	☐	For Bayesian analysis, information on the choice of priors and Markov chain Monte Carlo settings
☒	☐	For hierarchical and complex designs, identification of the appropriate level for tests and full reporting of outcomes
☒	☐	Estimates of effect sizes (e.g. Cohen's *d*, Pearson's *r*), indicating how they were calculated

Our web collection on statistics for biologists contains articles on many of the points above.

Software and code

Policy information about availability of computer code

Data collection	*Provide a description of all commercial, open source and custom code used to collect the data in this study, specifying the version used OR state that no software was used.*
Data analysis	*Provide a description of all commercial, open source and custom code used to analyse the data in this study, specifying the version used OR state that no software was used.*

For manuscripts utilizing custom algorithms or software that are central to the research but not yet described in published literature, software must be made available to editors and reviewers. We strongly encourage code deposition in a community repository (e.g. GitHub). See the Nature Portfolio guidelines for submitting code & software for further information.

Data

Policy information about availability of data

All manuscripts must include a data availability statement. This statement should provide the following information, where applicable:

- Accession codes, unique identifiers, or web links for publicly available datasets
- A description of any restrictions on data availability
- For clinical datasets or third party data, please ensure that the statement adheres to our policy

The RNA-seq data generated in this study have been deposited in the NCBI Gene Expression Omnibus under GE O accession GSE 220556. All remaining data can be found in the Article, Supplementary and Source Data files.

=

Research involving human participants, their data, or biological material

Policy information about studies with human participants or human data. See also policy information about sex, gender (identity/presentation), and sexual orientation and race, ethnicity and racism.

Reporting on sex and gender	*Use the terms sex (biological attribute) and gender (shaped by social and cultural circumstances) carefully in order to avoid confusing both terms. Indicate if findings apply to only one sex or gender; describe whether sex and gender were considered in study design; whether sex and/or gender was determined based on self-reporting or assigned and methods used. Provide in the source data disaggregated sex and gender data, where this information has been collected, and if consent has been obtained for sharing of individual-level data; provide overall numbers in this Reporting Summary. Please state if this information has not been collected. Report sex- and gender-based analyses where performed, justify reasons for lack of sex- and gender-based analysis.*
Reporting on race, ethnicity, or other socially relevant groupings	*Please specify the socially constructed or socially relevant categorization variable(s) used in your manuscript and explain why they were used. Please note that such variables should not be used as proxies for other socially constructed/relevant variables (for example, race or ethnicity should not be used as a proxy for socioeconomic status). Provide clear definitions of the relevant terms used, how they were provided (by the participants/respondents, the researchers, or third parties), and the method(s) used to classify people into the different categories (e.g. self-report, census or administrative data, social media data, etc.) Please provide details about how you controlled for confounding variables in your analyses.*
Population characteristics	*Describe the covariate-relevant population characteristics of the human research participants (e.g. age, genotypic information, past and current diagnosis and treatment categories). If you filled out the behavioural & social sciences study design questions and have nothing to add here, write "See above."*
Recruitment	*Describe how participants were recruited. Outline any potential self-selection bias or other biases that may be present and how these are likely to impact results.*
Ethics oversight	*Identify the organization(s) that approved the study protocol.*

Note that full information on the approval of the study protocol must also be provided in the manuscript.

Field-specific reporting

Please select the one below that is the best fit for your research. If you are not sure, read the appropriate sections before making your selection.

☒ Life sciences ☐ Behavioural & social sciences ☐ Ecological, evolutionary & environmental sciences

For a reference copy of the document with all sections, see nature.com/documents/nr-reporting-summary-flat.pdf

Life sciences study design

All studies must disclose on these points even when the disclosure is negative.

Sample size	No statistical methods were used to pre-determine sample sizes. Sample size for in vivo screening studies were empirically determined using barcode library recovery studies such that adequate coverage (>200 fold) of screening library could be achieved. Sample sizes for other in vivo experiments such as target validation, with or without use of the checkpoint inhibitors, was determined based on prior knowledge of typical biological variability with the tumor models and immunotherapy treatments.
Data exclusions	No data were excluded from the study.
Replication	Results presented in the manuscript were replicated at least twice in independent experiments or in two complementary tumor models. Results between replicates were reproducible.
Randomization	Allocation of experimental animals to the treatment groups were randomized to treatment condition(s) by cage following tumor onset. Covariant are not relevant to this study as isogenic mice with clean genetic background were used. In vitro experiments were performed using identical cellular preparations and were randomly assigned to the different experimental conditions.
Blinding	All tumor measurements conducted in this study were blinded to group allocation. All bioinformatics studies were automated and can be considered as blinded. The researchers conducting TCGA analysis of human PDAC samples and QRT analysis were blinded to group allocation. For experiments other than mentioned here, investigators were not blinded to treatment groups or genotypes, as knowledge of this information was essential to conduct the studies.

Reporting for specific materials, systems and methods

We require information from authors about some types of materials, experimental systems and methods used in many studies. Here, indicate whether each material, system or method listed is relevant to your study. If you are not sure if a list item applies to your research, read the appropriate section before selecting a response.

Materials & experimental systems

n/a	Involved in the study
☐	☒ Antibodies
☐	☒ Eukaryotic cell lines
☒	☐ Palaeontology and archaeology
☐	☒ Animals and other organisms
☒	☐ Clinical data
☒	☐ Dual use research of concern
☒	☐ Plants

Methods

n/a	Involved in the study
☒	☐ ChIP-seq
☒	☐ Flow cytometry
☒	☐ MRI-based neuroimaging

Antibodies

Antibodies used	The following primary antibodies were used in this study: Anti-Cytokeratin 19 antibody [RCK108] (1:200, Abcam ab9221), Anti-USP15 monoclonal antibody (M01), clone 1C10 (1:500, Novus Biological H00009958-M01), Anti-GAPDH (6C5) (1:1000, Santa Cruz sc-32233), p21 (1:200, Santa Cruz sc-6246), P53 (1:500, Santa Cruz (DO-1): sc-126), MDM2 (1:200, Santa Cruz sc-965), NRF2 (1:500, Cell signaling D1Z9C).
Validation	Antibodies were validated as described at the Manufacturers webpage. Antibodies against USP15 and p53 were further validated in the Schramek lab using isogenic knock-out cell lines. MDM2 and NRF2 antibody was validated in the Schramek lab using specific pathway agonists to stabilize their expression.

Eukaryotic cell lines

Policy information about cell lines and Sex and Gender in Research

Cell line source(s)	Primary mouse tumor KPC and KC cells were derived in the Schramek lab from the corresponding genetically engeneered mouse model. Human PANC1 and MiaPaCa-2 cell lines were obtained from ATCC.
Authentication	The PANC1 and MiaPaCa-2 cell lines were obtained from ATCC collection and identification as well as authentication was performed by company.
Mycoplasma contamination	All cell lines were regularly tested for mycoplasma and confirmed to be negative.
Commonly misidentified lines (See ICLAC register)	No commonly misidentified cell lines were used in this study.

Animals and other research organisms

Policy information about studies involving animals; ARRIVE guidelines recommended for reporting animal research, and Sex and Gender in Research

Laboratory animals	B6.FVB-Tg(Pdx1-cre)6Tuv/J and B6.129-Krastm4Tyj and 129S4-Trp53tm3Tyj and R26-LSL-Cas9-GFP [#026175 in C57/Bl6 background were obtained from the Jackson laboratories. conditional USP15-tm1c [C57BL / 6N-Usp15tm1c(EUCOMM)Wtsi / Tcp] were kindly provided by Philippe Gros.
Wild animals	There was no wild animals used in this study.
Reporting on sex	Equal numbers of male and female animals were used throughout the study without any bias. Sex stratified analysis was not performed as sample size is too small.
Field-collected samples	Samples were not collected from the field
Ethics oversight	Animal husbandry, ethical handling of mice and all animal work were carried out according to guidelines approved by Canadian Council of Animal Care and under protocols approved by the Centre for Phenogenomics Animal Care Committee (18-0272H).

Note that full information on the approval of the study protocol must also be provided in the manuscript.

Plants

Seed stocks	NA
Novel plant genotypes	NA
Authentication	NA
