# Supplementary material for: In vivo CRISPR screens reveal SCAF1 and USP15 as drivers of pancreatic cancer
Source: Nat Commun. 2024 Jun 20;15:5266. doi: 10.1038/s41467-024-49450-3 (PMC11189927; doi:10.1038/s41467-024-49450-3)
Supplement: Supplementary file 4 — Description of Additional Supplementary Files [file 41467_2024_49450_MOESM4_ESM.pdf]

Supplementary Data files 1. PDAC sgRNA library and non-targeting library.

List of sgRNAs targeting pancreatic cancer long tail genes ordered as a pooled oligo chip (CustomArray Inc., USA) and cloned into AAV sgRNA-H2B-RFP engineered from AAV:ITR-U6-sgRNA(backbone)-pCBh-Cre-WPRE-hGHpA-ITR kindly provided by Feng Zhang (Addgene plasmid #60229). PDAC library sgRNAs were obtained from Hart *et al.*,[71] (4 sgRNAs/gene) and non-targeting sgRNAs were obtained from Sanjana *et al*.,[72]

Supplementary Data files 2. Candidate genes

List of identified PDAC driver genes with enriched sgRNAs in tumor DNA obtained from the PDAC mouse model (targeted by ≥2 sgRNAs) , in multiple tumors and/or metastatic foci.

Supplementary Data files 3. DEG and GSEA

Differentially expressed genes (DEG) (false discovery rate (FDR, Benjamini-Hochberg) < 0.05 and absolute log2 fold-change > 1), and Gene set enrichment analyses (GSEA) of sgUsp15 and sgScaf1 compared to KC cells sgCtrl.

Supplementary Data files 4. GSEA olaparib

Gene set enrichment analyses (GSEA) of sgUsp15 and sgScaf1 compared to KC cells sgCtrl upon Olaparib treatment.

Supplementary Data files 5. Primers – gRNA

List of sgRNAs and primers used in the manuscript.
